# The National Medical Society of the District of Columbia

**Published:** 1870-01

**Authors:** 


### The National Medical Society of the District of Columbia.

Through the kindness of our friend Dr. Toner, we have received copies of several reports, one of which is that of the establishing of a new Medical Society, of which Dr. Robert Reyburn was chosen president, and Dr. Gray secretary. The occasion of the forming of a new society was the refusal of the old society to admit, on any consideration, colored physicians as members. The National Society propose to admit qualified physicians to membership to exclude irregular practitioners and to adopt a revised code of ethics.

